# Establishing of cancer units in low or middle income african countries: angolan experience - a preliminary report

**DOI:** 10.11604/pamj.2014.19.291.5320

**Published:** 2014-11-17

**Authors:** Fernando Miguel, Ana Vaz Conceição, Lygia Vieira Lopes, Dora Bernardo, Fernando Monteiro, Fernanda Bessa, Cristina Santos, João Blasques Oliveira, Lúcio Lara Santos

**Affiliations:** 1 National Oncology Centre, Luanda, Angola; 2 Cancer Unit of Girassol Clinic, Luanda, Angola; 3 Cancer Unit of Sagrada Esperança Clinic, Luanda, Angola; 4 Portuguese Institute of Oncology, Porto, Portugal; 5 ONCOCIR- Education and Care in Oncology, Angola; 6 Experimental Pathology and Therapeutics Group and Surgical Oncology Department, Portuguese Institute of Oncology, Porto, Portugal

**Keywords:** Angola, establishing cancer units, assessment checklist

## Abstract

**Introduction:**

The number of cancer cases and related deaths worldwide is expected to double over the next 20-30 years. African countries will be the most affected by the burden of cancer. The improving economic situation of Angola creates conditions for an increase in life expectancy which by itself is associated with an increased risk of oncological diseases. Because cancer therapy requires a multidisciplinary approach, trained health professionals, satisfactory infrastructure and appropriate facilities, the availability of effective cancer therapy is a difficult task that requires support. The aim of this article is to share our experience achieved in the establishment of cancer units in Angola and to validate our checklist for this action.

**Methods:**

The survey method was a questionnaire addressed to Angolan cancer units, in order to evaluate the usefulness and feasibility of a checklist developed by the authors - *The Cancer Units Assessment Checklist for low or middle income African countries* - which was used previously in the establishment of those units. Afterwards, the crucial steps taken for the establishing of the main sites of each cancer unit considering, facilities, resources and professionals, were also recorded.

**Results:**

All cancer units reported that the checklist was a useful tool in the development of the cancer program for the improvement of the unit or the establishing of cancer unit sites. This instrument helped identifying resources, defining the best practice and identifying barriers. Local experts, who know the best practices in oncology and who are recognized by the local heads, are also important and they proved to be the major facilitators.

**Conclusion:**

The fight against cancer has just started in Angola. The training, education, advocacy and legislation are ongoing. According to our results, the assessment checklist for the establishment of cancer units is a useful instrument.

## Introduction

In the 2014 World Cancer Day the WHO African Regional Director said: “Every year, nearly 8 million people die of cancer but many of these deaths can be avoided with greater public awareness, increased government support and funding for prevention, detection and treatment. Cancer is not a disease affecting the affluent and elderly people, and developed countries alone. It is a global epidemic, affecting all ages, in low, middle and high income countries. The number of cancer cases and related deaths worldwide is expected to double over the next 20-30 years. African countries will be the most affected by the burden of cancer, but are of all developing countries the least able to cope with the challenges cancer presents”. In this sense it is very important to build the capacities for cancer control [1].

Cancer control is understood as the main public health action designed to reduce incidence and mortality as well as to improve the quality of life of patients. It also includes the systematic implementation of evidence-based strategies for: prevention; early detection, diagnosis, treatment and palliative care.

In the fifty-seventh session of WHO Regional Committee for Africa held in Brazzaville, at 27–31 August 2007 the attendees underlined that most countries in Africa do not have the satisfactory infrastructure and facilities for cancer treatment, which includes surgery, chemotherapy and radiotherapy. Because cancer therapy requires a multidisciplinary approach, trained health professionals, satisfactory infrastructure and good facilities, the availability of effective cancer therapy is often an unrealistic objective [2].

However, in the last seven years, according to this point of view, several African countries have developed serious approaches in creating resources to treat cancer patients. Angola is one of these countries [3].

Since 2010, using 2008 Angola Globocan data, the National Health Plan and international published advocacy, the health authorities started to develop a strategy for cancer control. Meanwhile our research team identified existing resources for cancer diagnosis and treatment and described the needs, in order to help the development of cancer units in Angola [4]. Based on these data, we drew up a checklist form, *The Cancer Units Assessment Checklist for low or middle income African countries*, which we will address in this article.

Angola pointed out their Health Plan, entitled PNDS (Plano Nacional de Desenvolvimento Sanitário), to outline a more specific set of priorities over a medium-term. The PNDS adopted new incentives for health sector professionals; implemented new technologies, acquired adequate and sustainable finance and a modern and efficient management system. These priorities will be achieved through 47 ‘projects’ that address the technical and administrative challenges affecting the Angolan health sector today. The 14^th^ project is dedicated to cancer control and addresses the establishing of cancer units [5].

A cancer unit is a health facility that is capable of managing patients within a defined range of cancer, according to contemporary standards of good practice. The establishing of cancer units lies at the heart of successful application of this policy; it is not simply an exercise in the rebadging of existing institutions. The establishing of cancer units is a prospective process which requires the introduction of changes within a set timescale in clinical organization and practice but also in the management process of the health unit involved. Services offered by these units should reach identified standards such as conforming to established guidelines and participating in externally run audit programs [6].

Buonaguro FM et al. underlined the lack of robust data from well-designed and well-conducted studies about establishing cancer units in resource-limited settings, which complicates forecasting [7].

The aim of this article is to share the experience achieved in the establishment of cancer units in Angola and to perform the validation of *The Cancer Units Assessment Checklist for low or middle income African countries* for this action.

## Methods

This research was conducted between January 2012 and January 2014 in Angola. It used as survey method an assessment questionnaire developed by the authors - *The Cancer Units Assessment Checklist for low or middle income African countries* which was addressed to several hospitals in Angola. This instrument was applied in order to answer questions like: *which kind of cancer should a specific unit treat and what is needed to establish a cancer unit?* It also evaluated its validity. This questionnaire was based on the Questionnaire Quality standards of Organization of European Cancer Institute's and Association of Community Cancer Centers Guidelines, since there are no tools developed and adapted to African realities [8, 9]. The checklist assesses 10 different domains and includes the following main questions that should be answered with *Yes, Partially, No or Not Applicable:* 1)policy and Cooperation: is there an oncologic and cooperation plan in place? Are there national and international partners involved in this plan? 2) cancer data registration: is there a hospital, pathology and population based-cancer registry? 3) accuracy of the diagnostic services: is there a regular audit system about radio-diagnosis and imaging, pathology and laboratory accuracy? 4) responsibilities and tasks of the oncology team: is there a clear definition of the roles and tasks? 5) good clinical practice: is there an integrated care, selection criteria, registration and evaluation of recommendations to the multidisciplinary oncology team meeting, that includes compliance with guidelines? 6) Cancer treatment process: are there certified physical facilities and equipment for drug preparation (centralized unit) and infusion, surgical and radiation treatments? Is there a written procedure for acquisition, preparation, protocols prescription and administration of cytotoxic drugs? Is there an ambulatory oncology service, ward facilities, protocol assuring continuity of care and treatment complications registry? 7) safeguarding the quality: Is there a quality assurance in all areas, risk management and safety requirements? Is there a patient satisfaction inquiry? A periodical policy review and control of toxic waste is being performed? 8) patient and family support services: Are there rehabilitation, pain management, palliative care and psycho-oncology services? Are patients navigation, social counseling and family involvement in care implemented? 9) teaching, continuing education and research: Does the institution screen the training needs to define an educational program? Is there participation in oncology teaching and clinical research? 10) removing barriers and promoting stakeholders engagement: is there a systematic process to identify the stakeholders and barriers against the establishment of cancer units? The hospitals involved in this survey were:

*The National Oncology Centre* (NOC) in Luanda, the only public cancer specialized hospital, is the main center for the treatment of cancer patients in Angola. This hospital performs diagnosis, surgery, chemotherapy and radiation treatment and receives the people of the lowest income - which are the majority - treated for cancer in Angola.

*Girassol Clinic* (GC), a private clinic that belongs to the public company Sonangol and is able to perform diagnosis, surgery and radiotherapy. This unit treats Sonangol′s employees and some of the wealthiest Angolans.

*Sagrada Esperança Clinic* (SEC), a private clinic that belongs to a public company named Endiama and performs cancer diagnosis and surgery. SEC serves an intermediate segment of patients in terms of income - usually local and expats with health insurance plans and Endiama′s employees.

We evaluated and recorded the activities undertaken to improve conditions at the NOC and to establish GC and SEC cancer units, according to our checklist. Documents were required to support the given answer on each specific question to the participating units. According to the answers received, an action plan should be developed taking into account what actions or changes will occur, who will carry out these changes, when they will take place and what resources are needed.

In each participating hospital, the most prevalent cancers, the crucial steps developed for the establishing of cancer unit's sites, facilities, professionals and resources, were recorded.

## Results

Two years after the start of this study the profile of the units developed is as follows:

### National Oncology Centre

**Figure 1:**
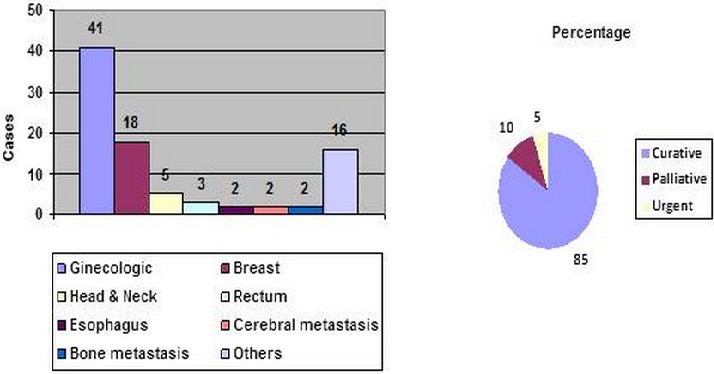
Number of patients treated with radiation therapy in NOC and treatment profile in 2013

Each year about a thousand new patients were admitted (Table 1), assessed within a multidisciplinary approach and it was possible to start treating patients with radiotherapy (Figure 1). The action protocols for the most prevalent oncological pathologies were defined and shared with both emerging units under a cooperation program. The facilities resources including inpatient wards and surgical areas are increasing. NOC was an active member in the drafting of National Project no 14, dedicated to cancer control. It is also involved in the information to the public about oncological diseases.

**Table 1:** Number of patients admitted and treated in NOC (without radiation), GC and SEC (2013) according hospital registry and diagnosis confirmed

Cancer Site	NOC	GC	SEC
Breast	314	45	25
Cervix	180	5	9
Prostate	101	14	12
Esophagus	25	9	4
Stomach	42	28	7
Colorectal	25	6	13
Lung	11	5	9
Kaposi	65	-	2
Sarcoma	45	-	5
Hematologic	92	44	6
Others	429	27	55

The implementation of our checklist allowed NOC to reevaluate their practice and develop a set of priorities for future activity, among which we emphasize: the improvement in the accuracy of the diagnostic services (imaging and pathological diagnosis and its interpretation), increasing radiotherapy facilities, improving safety and quality in the preparation of chemotherapy and starting the toxic waste control. They also defined a more proficient framework for its national and international collaborators. NOC intends to participate in the effort of oncology training in health schools and clinical research. A hospice program is not yet defined.

### Girassol Clinic

**Figure 2:**
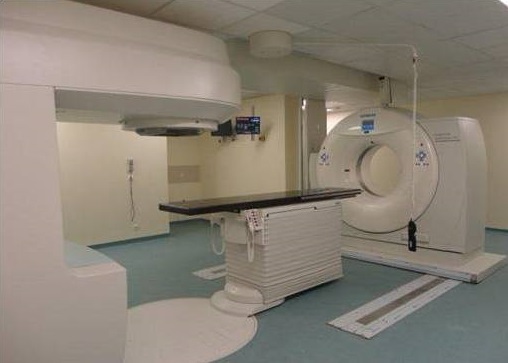
Radiotherapy facilities in GS

The building construction plan and equipment acquired for this Clinic considered as a priority and since the beginning, the existence of a cancer unit (radiotherapy, nuclear medicine, chemotherapy preparation and ambulatory infusion areas, operating theater and inpatient ward). Construction and supervision of radiotherapy and nuclear medicine had the support of the International Atomic Energy Agency (Figure 2). GC developed their own training plan for technical staff and the profile of consultants. This area is in full operation. The GC established multidisciplinary approach as a decision making process for the best treatment. Regarding the area of clinical and surgical oncology, GC assembled a team that included internal and external advisors. The establishing of the cancer unit was performed according to the checklist developed and with advisers of the Portuguese Institute of Oncology - Porto. Currently, GC treats about 200 patients per year (Table 1). During the period of construction and organization of chemotherapy preparation and infusion area, which will start working soon, GC had the support of NOC in accordance with a cooperation protocol previously established. A home care program for patients in palliative setting was developed and is fully implemented.

### Sagrada Esperança Clinic

SEC decided recently to build a cancer unit, given the increasing number of cancer patients seeking treatment per year (Table 1). The first activity was to create a hospital-based cancer registry and to develop a program for early diagnosis of cancer for Endiama's employees. With the support of ONCOCIR-Education and Care in Oncology - Angola and using the authors checklist the plan for the future cancer unit and their scope, was designed. SEC also conducted the training of the oncology team. Based on this definition and in accordance with existing resources in neighboring hospitals, the needed resources, were established. A protocol treatment was signed with NOC and Portuguese Institute of Oncology - Porto during the period of establishment of the cancer unit, in order to ensure access oncological treatment to the SEC cancer patients. Data from the SEC cancer registry showed a huge number of patients with advanced cancer at diagnosis, prompting the SEC oncology team to design and implement the first national course on palliative care. The accuracy of the diagnostic services was also reassessed. A dedicated building is currently in constructions, where the preparation and infusion chemotherapy unit and palliative care service will be hosted. Improvement of safety and control of biohazard and the discarding and destruction of chemotherapy waste, were a major concern. For that purpose SEC acquired the appropriate technology resources which may help others cancer units in this field. Each hospital acknowledged that the author's checklist was used for the action of establishing their cancer unit plan.

## Discussion

Cancer control in low-and-middle income countries (LMIC), is generally characterized by a complex program involving joint actions for cancer prevention, information, early diagnosis, treatment and palliative care. All this occurs in an environment where infectious diseases are prevalent; health professionals are still in training and monetary resources are tight. Additionally there has been a lack of cancer information in LMIC which in partly due to not having cancer registries and a vital statistics systems properly implemented.

In Angola as in the UK, the main reasons to define where cancer units should be located are: the hospital scope, patients access, expertise, potential work volume by cancer location, capability, local resources and financing [6]. Until now, in all hospitals studied, mostly adults were treated. Cancer registry and the checklist for action are a good help to answer these questions in order to establish a proficient cancer unit.

### Cancer registry

In June 2012, Angola imPACT mission, implemented by Programme of Action for Cancer Therapy (PACT) of the International Atomic Energy Agency (IAEA), produced recommendations in order to improve population-based cancer registration. To accomplish this, the NOC organized and sponsored a course entitled ‘Cancer Registration – Principles and Methods, held in Portuguese and carried out at the NOC facilities that included GC and SEC technicians. Currently, only NOC, GC and SEC, aggregate data of patients diagnosed and treated for cancer in their hospital-based cancer registries. The old pathology-based registry from Américo Boavida Hospital is still active. However these registries are fragile, fragmented and in need for better quality control. Therefore, population-based cancer registry in Angola is an imperative need.

### Checklist for action and external advisers

There are no validated instruments to assist the establishing of cancer units in LIMC. Several organizations have developed knowledge and useful guidelines in order to assist hospitals in this task, but often only applied to a single disease like breast cancer [10]. Another source of help and knowledge are the guidelines for certification of cancer units such as: ASCO′s Quality Oncology Practice Initiative (QOPI^®^), OECI, ACCC and Committee on Cancer Control in Low- and Middle-Income Countries of Institute of Medicine (US) [8, 9, 11, 12]. However, there are no reports of using these guidelines in establishing oncology units in LIMC. For this reason we built the action checklist previously described. According to our survey, conducted in 3 hospitals, the usefulness of this checklist has been proven. The consultants opinions, based on their own experience, also had an undeniable value. However, we emphasize that consultants should take into account local idiosyncrasies. Local experts in oncology, that are recognized by their fellow colleges and local heads, are the major facilitators.

Therefore, we consider that the utilization of this systematized tool - the assessment checklist - associated consultants experience and the partnership with certificated cancer centers recommendations/guidelines must be taken into account. In this sense, there are models of collaborative partnerships between a combination of low, middle and upper-income countries’ cancer centers. These models could provide useful pointers for improving cancer prevention and control capacity, in low resource settings [13]. The assessment checklist will be filled in by the head of oncologic team with the support of others services involved as well as by the quality control department when available, or by the external advisers.

### Accuracy of the diagnostic services

An accurate diagnosis is the first step for an appropriate treatment plan, therefore, in this survey, we evaluated the cancer diagnostic services of NOC, GC and SEC. A critical lack of image-guided needle sampling of suspicious lesions and advanced pathology procedures in order to optimize systemic management, still remains as also mentioned by El Saghir NS et al. [14]. Additionally magnetic resonance imaging and diffusion analysis, when available, are important tools for the evaluation of cancer staging and treatment response, especially in middle and low income nations as Angola.

### Surgical therapy

The ability to perform oncologic surgery is the mainstay of locoregional treatment. Surgical training in LMICs is fundamental, since surgeons have less experience to perform these procedures. Increasing access to quality cancer surgery is crucial [15]. According to our survey it is essential to develop training programs in surgical oncology to harmonize the knowledge and improve the quality of cancer surgery.

### Radiation oncology

The availability of radiation therapy has a major impact on local tumor control for early and locally advanced disease. Effectiveness and safe radiation therapy can also improve overall survival rates. GS cancer unit has one of the most advanced radiotherapy cancer treatments available, including intensity-modulated radiation therapy (IMRT) and braquitherapy and NOC has a Variant^®^equipment. Both institutions have doctors and radiotherapy technicians working full-time. The patients treated with radiotherapy are increasing.

### Nuclear medicine

According to Gholamrezanezhad A et al. nuclear medicine is a routine and a vital part of diagnosing and treating many disorders and diseases including cancer and may play an important role also in LMIC. [16]. GS has a nuclear medicine service that supports other cancer units.

### Systemic therapy and drug delivery

The use of systemic cytotoxic chemotherapy is effective in cancer treatment especially in locally advanced or metastatic tumors. The most common side-effects of chemotherapy are mucositis, nausea, vomiting, myelosuppression, infertility and alopecia. This treatment is carried out in an environment where infectious diseases are prevalent and where sanitary conditions are poor. Thus, the risk of complications is high and the organization should take into account the need for a close surveillance of febrile episodes. An effective pre and post medication and proper communication between patients and the cancer unit's multidisciplinary team must be ensured. The possibility of admission to the inpatient ward should be taken into account as well as a fast-track access to care for the medical oncologist. The flowcharts that have been used in Angola include this reality.

When the cost of cancer treatment is high, usually the patient decides not to continue or even not doing it at all. The affordability of cancer drugs is still a problem to be solved. The Ministry of Health attempted to minimize this problem giving free access to drugs in public facilities, but the increased number of cancer patients threatens the sustainability of this decision and creates some obstacles to its functionality. The 8^th^ Section of the latest WHO Essential Medicines List has been a great help in the purchasing of cancer drugs [17]. We defend the opportunity to provide the drugs included in a given protocol jointly, reducing actual difficulties when some component is not available.

Pharmacy services are responsible for drug therapy, from procurement to final disposition and documentation. A pharmacist should be able to do the cancer teaching program, either as an employee, or as a consultant to train, certify, prepare, validate medical prescription or advise the staff responsible for Pharmacy Services. All staff involved in the receipt of cancer therapeutic agents will be trained for all appropriate procedures, including the event of breakage, leakage, or other product damage. Preparation policies and procedures include: an appropriate environment, employee garb, instruction, work process, and environmental testing that conform to current international regulations. Established and available standards for dilution of solutions, concentrations, compatibility, and stability, along with current reference information. Disposal procedures for syringes, needles, and chemotherapy waste that conform to Angolan policies and international regulatory agencies should be implemented [9]. In order to fulfill these recommendations, the training of pharmacists and pharmacy technicians, the creating of clear rules, the verification of compliance and the construction of an appropriate environment including pharmaceutical storage have been taking place.

### Palliative care

Palliative care is still very unsatisfactory, although the national cancer control plan (14^th^ project) already includes palliative care and end-of-life care (e.g., morphine was added to the National Essential Medicines List). The Ministry of Health as well as the mentioned hospitals are currently exploring the feasibility of training health workers for the provision of palliative care in HIV patients, cancer patients as well as for other chronic diseases.

## Conclusion

The fight against cancer has just started in Angola. The Training, education, and legislation are ongoing. According to our results the assessment checklist for the establishing of cancer units is a useful tool in African LMIC. The evaluation of the progress of these units will only be conducted two years after their full operation.
